# Cytokines from parasites: manipulating host responses by molecular mimicry

**DOI:** 10.1042/BCJ20253061

**Published:** 2025-04-29

**Authors:** Rick M. Maizels, Henry J. McSorley, Hermelijn H. Smits, Peter ten Dijke, Andrew P. Hinck

**Affiliations:** 1 School of Infection and Immunity, University of Glasgow, Glasgow, U.K; 2 Division of Cell Signalling and Immunology, School of Life Sciences, University of Dundee, Dundee, U.K; 3 Department of Immunology, Leiden University Medical Center, Leiden, Netherlands; 4 Oncode Institute and Department of Cell and Chemical Biology, University of Leiden, Leiden, Netherlands; 5 Department of Structural Biology, University of Pittsburgh School of Medicine, Pittsburgh PA 15260, U.S.A.

**Keywords:** CD44, cytokines, evolution, immunomodulation, mimicry

## Abstract

Helminth parasites have evolved sophisticated methods for manipulating the host immune response to ensure long-term survival in their chosen niche, for example, by secreting products that interfere with the host cytokine network. Studies on the secretions of *Heligmosomoides polygyrus* have identified a family of transforming growth factor-β (TGF-β) mimics (TGMs), which bear no primary amino acid sequence similarity to mammalian TGF-β, but functionally replicate or antagonise TGF-β effects in restricted cell types. The prototypic member, TGM1, induces *in vitro* differentiation of Foxp3^+^ T regulatory cells and attenuates airway allergic and intestinal inflammation in animal models. TGM1 is one of a family of ten TGM proteins expressed by *H. polygyrus*. It is a five-domain modular protein in which domains 1–2 bind TGFBR1, and domain 3 binds TGFBR2; domains 4–5 increase its potency by binding a co-receptor, CD44, highly expressed on immune cells. Domains 4–5 are more diverse in other TGMs, which bind co-receptors on cells such as fibroblasts. One variant, TGM6, lacks domains 1–2 and hence cannot transduce a signal but binds TGFBR2 through domain 3 and a co-receptor expressed on fibroblasts through domains 4–5 and blocks TGF-β signalling in fibroblasts and epithelial cells; T cells do not express the co-receptor and are not inhibited by TGM6. Hence, different family members have evolved to act as agonists or antagonists on various cell types. TGMs, which function by molecularly mimicking binding of the host cytokine to the host TGF-β receptors, are examples of highly evolved immunomodulators from parasites, including those that block interleukin (IL)-13 and IL-33 signalling, modulate macrophage and dendritic cell responses and modify host cell metabolism. The emerging panoply and potency of helminth evasion molecules illustrates the range of strategies in play to maintain long-term infections in the mammalian host.

## Parasite immune evasion

Pathogens that establish chronic infections in their host must first evade the immune responses designed to lyse, kill, incapacitate, or eject them. Micro-organisms can take the approach of outpacing immunity and undergo rapid selection of escape mutants, while protozoal parasites can vary their surface-exposed antigens, such as with trypanosomes [1,2], rendering the antibody response raised against them to be non-functional. This strategy is not available to slower growing helminth macro-parasites, which do not proliferate in their hosts and, therefore, cannot rapidly select for advantageous mutations. Hence, many parasites take alternative routes through transition into different life stages and/or by releasing immunomodulatory factors, which tailor the immune response to their benefit [3]. Such sophisticated immunomodulation can result in a tolerance to the parasite, which at best can benefit both the parasite and host by minimising collateral damage, immune hyper-responsiveness and enabling asymptomatic chronic infection. More detrimentally, helminth infection can result in immune suppression, co- or hyper-infection, cancer progression/metastasis and reduced vaccination protection against other pathogens [4–6].

In viral infections, immune evasion factors are well understood and shown to suppress critical pathways, such as major histocompatibility complex class I antigen presentation at virtually every step in the process. In contrast, helminth immunomodulation has only begun to be understood with the recent expansion of molecular studies on these parasites. A broad range of mediators have now been discovered [7–11], which either block anti-helminth immune responses or induce immune tolerance depending on their life stage or tissue residence.

## Transforming growth factor-β – a pivotal cytokine

Cytokines are soluble messenger proteins for intercellular communication that activate and regulate various aspects of immunity, inflammation and tissue homeostasis [12–15]. The complexity of the cytokine network reflects the diverse nature of pathogens, each requiring a tailored immune response. Interestingly, redundancy within this network may have arisen from pathogens attempting to disrupt cytokine functions. This review focuses not on the role of cytokines during an infection but rather on how parasites alter the host cytokine network to their benefit.

A particularly intriguing study area is the hijacking of transforming growth factor-β (TGF-β) signalling by secreted parasite cytokines, exploiting host cell surface TGF-β serine/threonine kinase receptors (TGFBR1 and TGFBR2). TGF-β is an exceptional cytokine with functions far beyond the immune system, playing crucial roles in development, cellular metabolism, tissue homeostasis and repair [16,17]. Within the immune system, TGF-β is largely viewed as an anti-inflammatory cytokine through its induction of regulatory T cells, but in parallel, it acts to drive class switching and differentiation of IgA^+^ B cells and modifies dendritic cell (DC) and macrophage phenotypes [18]. The critical nature of TGF-β-mediated immune regulation is highlighted by the early mortality of mice lacking TGF-β1, through the development of multi-organ inflammatory disease, particularly in the intestinal tract [19]. Due to the critical role of TGF-β signalling in tissue differentiation and formation, mice lacking the other isoforms (TGF-β2 and TGF-β3) show congenital developmental defects [20,21], and animals deficient in TGF-β receptor II (TGFBR2) suffer embryonic or perinatal mortality [19].

While TGF-β is critical for survival in mammals, it poses threats — especially in cancer [22]. The tumour microenvironment often contains elevated levels of TGF-β, which has been found to block T cell activation during checkpoint immunotherapy [23,24]. As a potent immune suppressor, TGF-β is also detrimental in various infectious settings [25], particularly in parasitic infections. This was first reported in the moderation of T cell responses to the river blindness nematode *Onchocerca volvulus* [26], and in the high levels of serum TGF-β in patients with Chagas’ disease, caused by the protozoan *Trypanosoma cruzi*, linked to the development of myocardiopathy [27].

In experimental animal models, an evident role for TGF-β was revealed upon infection by the murine intestinal helminth *Heligmosomoides polygyrus*, in which administration of the small molecule inhibitor SB431542 (that blocks the kinase activity of TGFBR1 and closely related receptors) accelerated worm expulsion [28]. Parallel results were obtained using the neutralising anti-TGF-β monoclonal antibody 1D11 in mice, significantly reducing infection with another intestinal parasite *Trichuris muris* [29]. *H. polygyrus* infection also elicits *in vivo* expansion of regulatory T cells that are required for parasite persistence [30]. The ability of *H. polygyrus* to stimulate Tregs suggested that it may activate host TGF-β signalling, a proposition that was confirmed when secretions collected from the parasite *in vitro* (*H. polygyrus* excretory/secretory products, HES) were added to T cells and induced expression of transcription factor Foxp3. Induction was found to be inhibited by SB431542 [28], confirming that the parasite could directly address the TGF-β pathway. Similar TGF-β-like activity has been reported in excretory/secretory (ES) products of a related Trichοstrongylid nematode, *Teladorsagia circumcincta* [28] and in extracts of a rodent filarial nematode *Litomosoides sigmodontis* [31], but only in the case of *H. polygyrus* has the active principle been identified as described below.

## Identification of the TGF-β mimics from *H. polygyrus*

**Figure 1:**
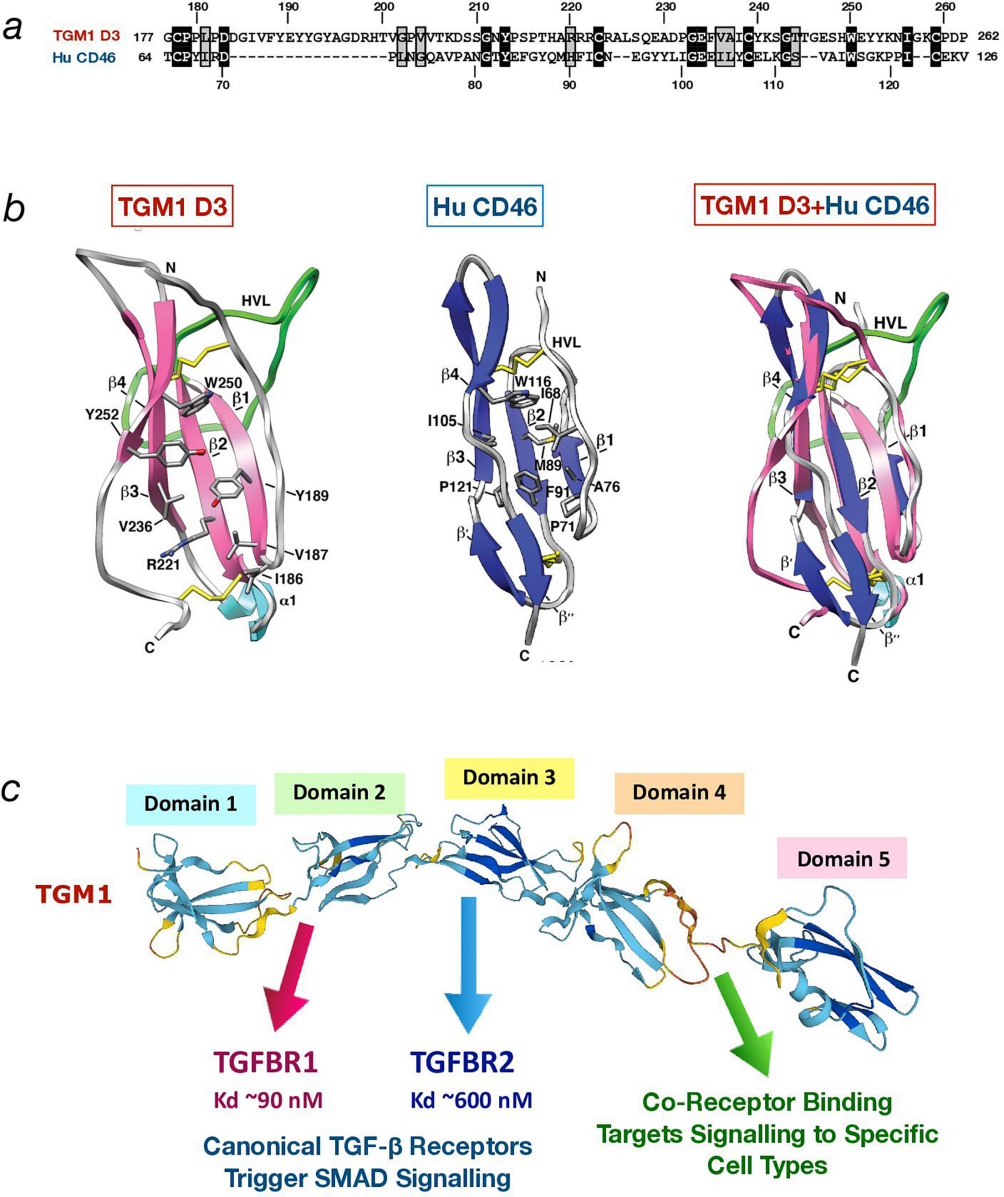
Sequence and structural characteristics of TGM1 and CCP proteins. (**a**) Alignment of TGM1 D3 with domain 2 of human CD46 [36]. Identical residues are shown in inverse shading, similar residues in shaded boxes. Amino acid positions in the respective proteins are indicated above and below the alignment. (**b**) Structural comparison of TGM1 D3 [36], left, with domain 2 of human CD46 (PDB 1CKL), centre and overlay of both structures, right. N- and C-termini of the domains are indicated, and positions of key amino acids. The four β sheets are indicated, as is the α1 helis. HVL, Hyper-Variable Loop. This part of the figure is reproduced from Ref. 36 in the *Journal of Biological Chemistry*, in accordance with the republishing policy of the American Society for Biochemistry and Molecular Biology. (**c**) Alpha fold model of TGM1, indicating receptor specificities of the different domains, and the experimentally determined dissociation constants (*Kd*) for TGFBR1 and TGFBR2. Affinities of TGM4 and TGM6 for these receptors are given in Table 1.

Since HES is a complex mixture of many hundred soluble proteins released by *H. polygyrus*, this material was fractionated by ion exchange and size exclusion chromatography, and fractions were screened for TGF-β-like activity. Proteins in active fractions were identified by mass spectrometry reconciled to a transcriptomic database from different stages of the parasite [32,33]. Selected candidates were expressed in a mammalian cell system as recombinant proteins, one of which proved to be a highly potent activator of TGF-β-like signalling, both in triggering an intracellular effector SMAD3-dependent reporter in fibroblasts, and by the induction of Foxp3 in murine T cells [34]. Remarkably, this active protein does not share primary amino acid sequence similarity with any TGF-β family member, which includes three TGF-β isoforms, activins and bone morphogenetic proteins [35]. It is, however, distantly related to the Complement Control Protein (CCP) family, which, although barely apparent at the amino acid sequence level (Figure 1a), is confirmed by conservation of three-dimensional structure (Figure 1b) [36]. Interestingly, the parasite product has acquired several insertions of amino acid tracts that enlarge external loops compared with canonical domains, such as human complement receptor CD46 (Figure 1b).

Importantly, unlike the mature bioactive TGF-β protein – which consists only of the ~110 aa C-terminal domain of a longer precursor – the *H. polygyrus* protein contains five homologous but nonidentical domains each forming a self-contained module, connected by short connecting segments (Figure 1c). A further striking distinction is that TGF-β is synthesised as an inactive pre-protein and stored until activated by integrins or a proteolytic event and released from latency-associated partners [37,38]. In contrast, the helminth product is immediately competent as a full-length native protein, and it could be argued that the parasite needs no such restraint on the activity of its immune modulator. Thus, it was named TGM1 indicating that it mimics TGF-β without being an evolutionarily related homologue. As detailed below, the domains act as modular units, each conferring separate functional properties to the full-length protein.

**Figure 2:**
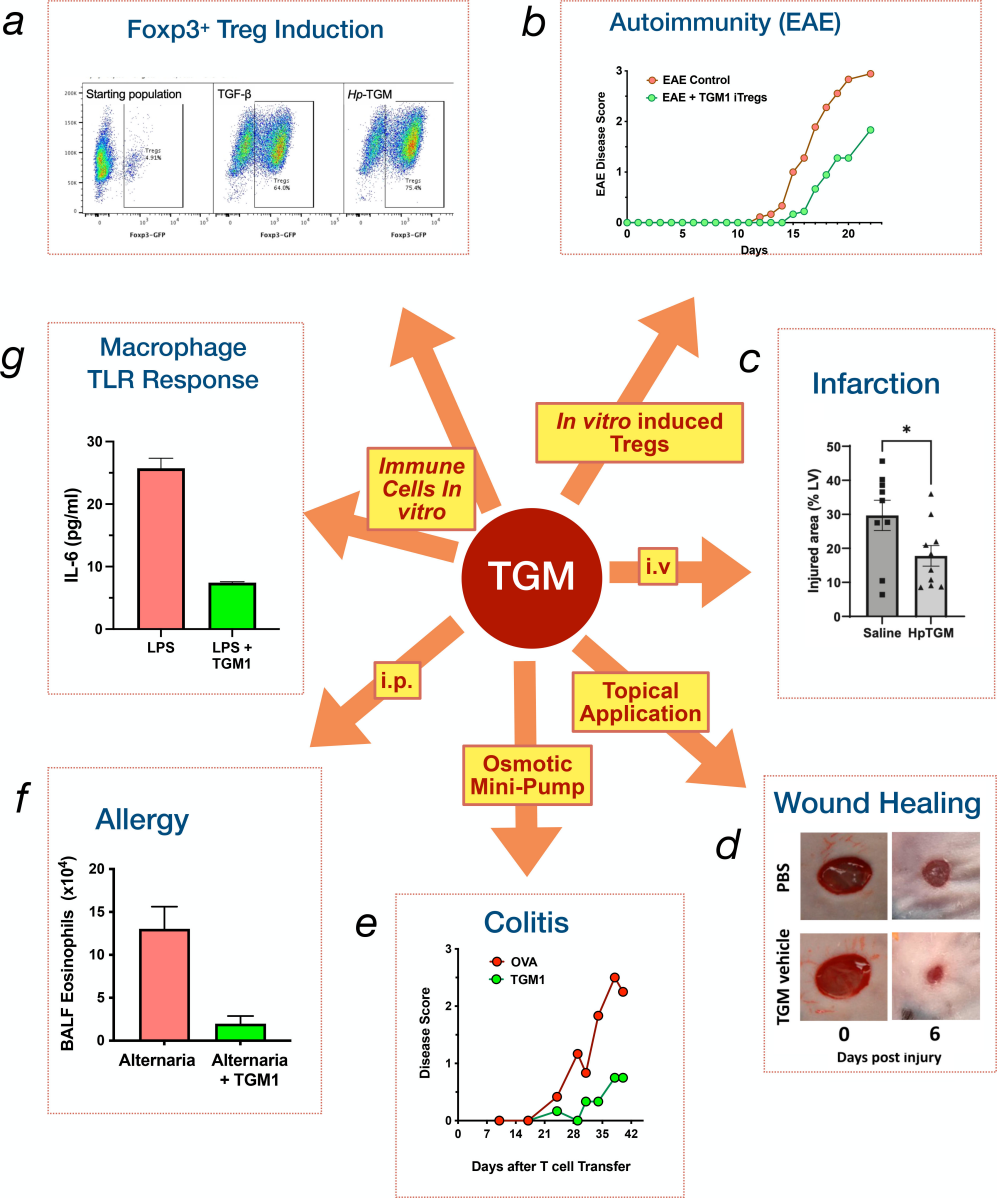
Immunological functions of TGM1 *in vitro* and *in vivo*. Schematic representing the various immune functions of TGM1. Clockwise from top left. (**a**) Induction of Foxp3 expression in murine [34,39 ] and human [40] T cells, representing the regulatory T cell (Treg) phenotype. (**b**) *In vivo* suppression of experimental autoimmune encephalomyelitis by adoptively transferred Tregs generated by *in vitro* treatment with TGM1, compared with TGF-β-induced Tregs [39]. (**c**) Reduction in cardiac injury during myocardial infarction by intravenous (i.v.) administration of TGM1 [39]. (**d**) Acceleration of skin wound healing in mice by topical application of TGM1 [41]. (**e**) Amelioration of colitis in RAG-deficient mice receiving Foxp3-negative T cells, in animals given subcutaneous osmotic minipumps releasing TGM1 [42]. (**f**) Suppression of allergic airway eosinophilic inflammation in bronchoalveolar lavage fluid (BALF) in mice receiving intranasal Alternaria alternata fungal allergen, and treated with intraperitoneal (i.p.) TGM1 [43]. (**g**) Inhibition of LPS-driven IL-6 release from murine bone marrow-derived macrophages in the presence of TGM1 [44]. TGF, transforming growth factor; TGM, transforming growth factor-β mimics.

TGM1 replicates the functional effects of TGF-β across a range of immune settings (Figure 2). For example, both murine [39] and human [40] T cells respond to TGM1 by inducing Foxp3 expression specifying Treg function. A closer comparison of the dose responses to the two ligands shows that while TGF-β activates fibroblasts at lower concentrations than TGM1, the latter is more potent than TGF-β at higher levels, displaying a steeper slope indicative of co-operativity in binding [39]. Similarly, SMAD2/3 phosphorylation in T cells is more rapid in response to TGF-β, but the slower response to TGM1 is sustained for longer [39]. As will be discussed below, the discovery of diverse co-receptor interactions with TGM1, but not TGF-β, may provide a mechanistic explanation for these observations.

Although Foxp3^+^ Tregs induced by TGF-β signalling are functionally immunosuppressive, they may not maintain this phenotype and can revert to a Foxp3^-^ effector phenotype, especially in an inflammatory environment [45]. However, a greater proportion of cells activated by TGM1 retain Foxp3 expression after transfer into mice with intestinal inflammation induced by dextran sodium sulphate, than is the case for TGF-β-induced Tregs [39]. TGM1 has a greater capacity to convert human Th17 effectors into Foxp3^+^ Tregs, arguing that memory as well as naïve T cells are converted into the regulatory compartment [40]. TGM1-induced Tregs are also fully functional with equivalent potency to TGF-β-induced Tregs when transferred to mice in an experimental autoimmune encephalomyelitis model [39]. Additionally, soluble TGM1 protein administered intraperitoneally protects mice against both airway allergy [43] and inflammatory colitis [42], and in the latter model, oral administration of TGM1 also attenuated disease [46], although in the *in vivo* setting, TGF-β signalling is likely to activate multiple cell types in addition to Tregs. In other analyses, TGM1 given intravenously reduced heart damage and scarring following myocardial infarction in mice [47], while when applied to skin wounds, it accelerated wound repair with a qualitative shift to scar-free healing that even renewed hair follicle formation [41].

**Figure 3:**
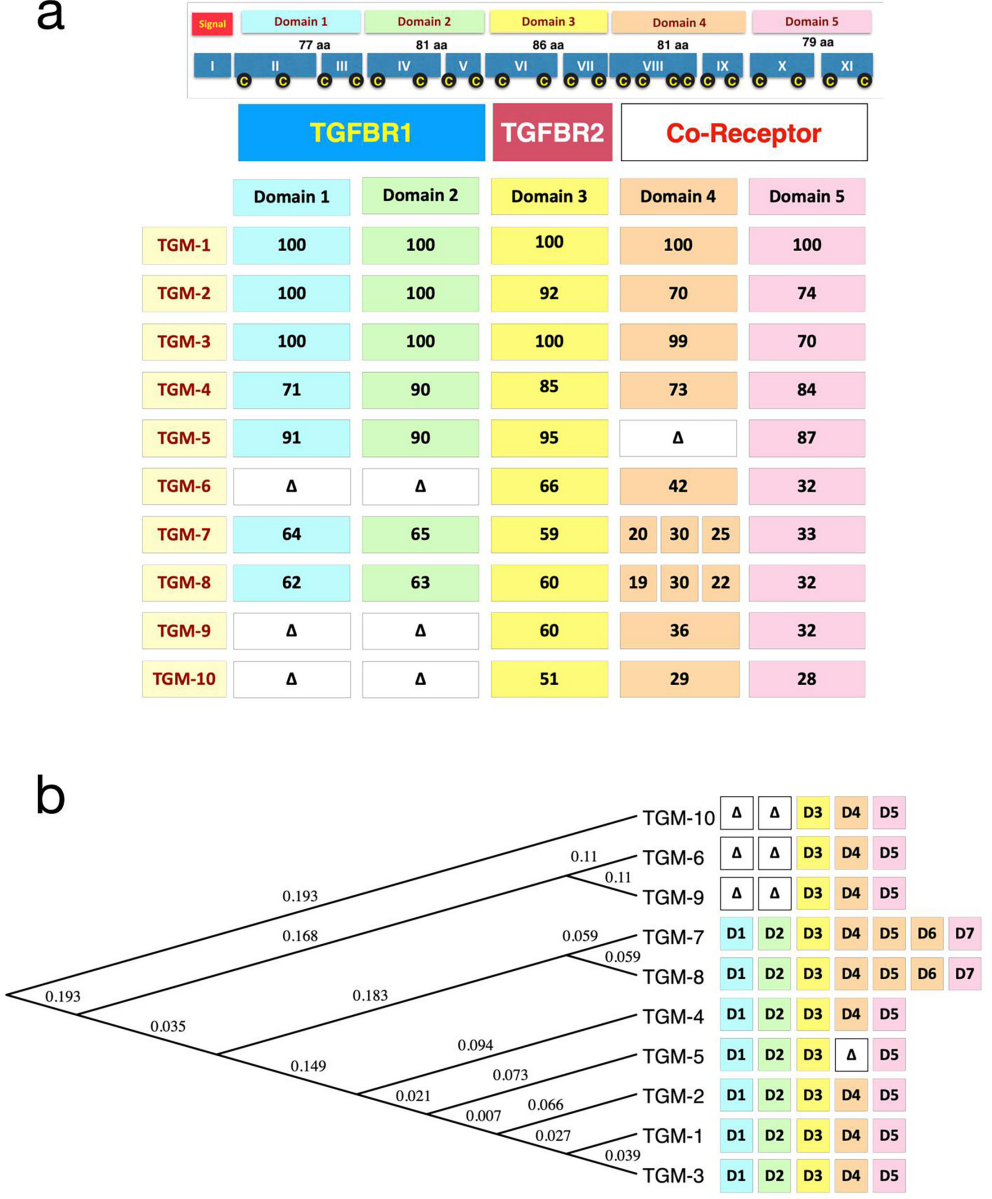
TGM family member domain organisation and phylogenetic relationship. (**a**) (Top section) Domain organisation of TGM1 and corresponding 11 exons in the *H. polygyrus* genome (Roman numerals); yellow C letters in black circles denote Cys residues. All disulphide bonds are intra-domain. (Main section) Organisation of domains in 10 family members, with percent amino acid identity of each domain to the corresponding domain of TGM1. Δ denotes absence of the domain(s). Note TGM7 and TGM8 have two additional domains, percentage identities shown are as indicated by colouring, with those with D4 of TGM1. Schematic is updated from Smyth et al [48] with corrected percent identities for D4-5 of TGM2 and the domains of TGM10 realigned. (**b**) Phylogenetic tree of the 10 full-length sequences of TGM family members. Numerals represent *P* values for branching topography. NB: For clarity, the TGM proteins are shown hyphenated (TGM-1), although normally the hyphen is omitted. TGF, transforming growth factor; TGM, transforming growth factor-β mimics.

Further investigation of the *H. polygyrus* secretome characterised by proteomic analysis of HES products [32,33] revealed a larger family of ten TGF-β mimic (TGM) proteins [48], (Table 1) comprising a variable number of homologous but non-identical domains (Figure 3a). Like other proteins, each domain contains at least two disulphide bonds that are fully conserved across all domains of each TGM family member; within the *H. polygyrus* genome, domains are each encoded by two exons with boundaries coinciding with domain junctions, indicative of an evolutionary module that can be duplicated and reduplicated over time. Indeed, a phylogenetic tree (Figure 3b) suggests that the three-domain proteins, TGM6, 9 and 10, are ancestral to the longer homologues, in line with earlier work indicating that domain (D)5 of TGM-10 may be the most primordial domain in the family [51]. Of note, TGM1–6 are predominantly produced by adult worms, and TGM7–10 by the tissue-dwelling larvae [33,52].

**Table 1:** Features of TGM family members.

		NCBI	MFB-F11 SMAD	T cell Foxp3	TGFBR1	TGFBR2	Co-receptor(s)	References
TGM1	422 aa SP + 5 domains	MG099712 AT059092	+++	+++	90 ± 1 nM	610 ± 10 nM	CD44 30 nM	[34,48]
TGM2	430 aa SP + 5 domains	MG429737 AVN88293	+++	+++	(100% identical to TGM-1)			[48] Ntang, E.Y.^1^
TGM3	429 aa SP + 5 domains	MG429738 AVN88294	+++	+++	(100% identical to TGM-1)	(100% identical to TGM-1)		[48]
TGM4	422 aa SP + 5 domains	MG429739 AVN88295	–	++	5.0 ± 0.1 nM	116 ± 2 µM	CD44 20 nM, CD49d CD206	[48,49]
TGM6	254 aa SP + 3 domains	MG429741 AVN88297	–	–	N/A (D1-2 are absent)	220 ± 100 nM	TBD : Not CD44	[48,50]
TGM7	599 aa SP + 7 domains	MG429742 AVN88298	–	–	Not tested	Not tested	TBD : Not CD44	[48]
TGM9	251 aa SP + 3 domains	MG429744 AVN88300	–	Not tested	N/A (D1-2 are absent)	Not tested	TBD : Not CD44	Mullin, T. and Singh, S.P.^1^
TGM10	251 aa SP + 3 domains	MG429745 AVN88301	–	Not tested	N/A (D1-2 are absent)	Not tested		Ciancia, C.^1^

TGM5 and TGM8 are not included as they have not been expressed or tested in experimental settings so far.

1 Unpublished studies.

SP+, predicted signal peptide (aa 1-18). TBD, to be determined.

## TGF-β receptor binding domains

**Figure 4:**
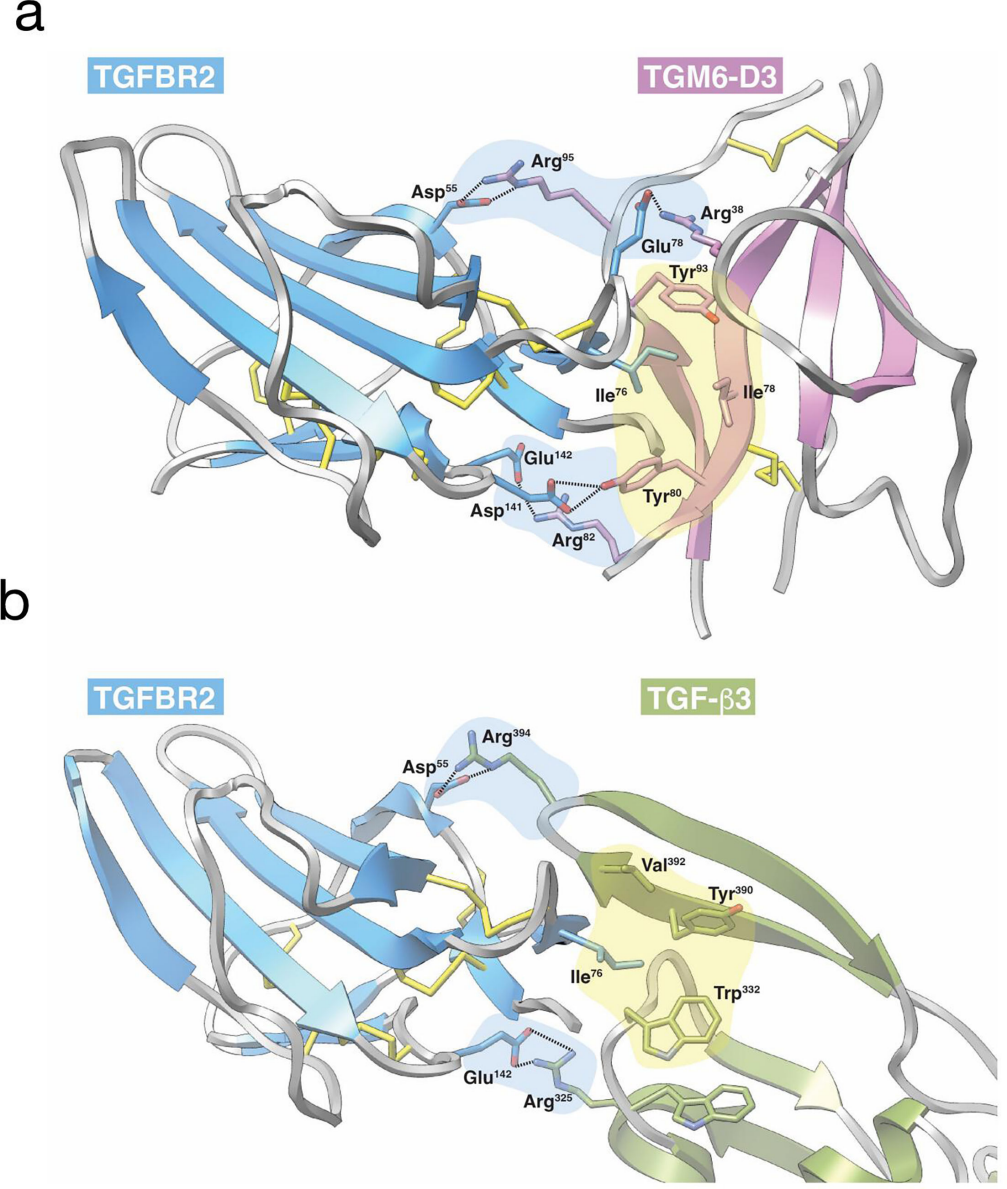
Mimicry of TGFBR2 binding by TGM domain 3. (**a**) Structure of TGM6-D3 (magenta) bound to the human TGFBR2 ectodomain (blue). Structure was determined using X-ray crystallography and was determined to a resolution of 1.4 Å [50]. Central hydrophobic interaction in which TGFBR2 Ile^76^ inserts into a pocket formed by TGM6-D3 Ile^78^, Tyr^80^ and Tyr^93^ is highlighted by yellow shading. Peripheral hydrogen-bonded ion pairs, TGFBR2 Asp^55^ and Glu^78^ with TGM6-D3 Arg^95^ and Arg^38^, respectively, and TGFBR2 Asp^141^ and Glu^142^ with TGM6-D3 Tyr^80^ and Arg^82^, respectively, that further stabilise the interaction and add specificity are highlighted by blue shading. (**b**) Structure of TGF-β3 (olive) bound to the human TGFBR2 ectodomain (blue). Structure was determined using X-ray crystallography and was determined to a resolution of 3.0 Å [53]. Central hydrophobic interaction in which TGFBR2 Ile^76^ inserts into a pocket formed by TGF-β3 Trp^332^, Tyr^390^ and Val^392^ is highlighted in yellow. Peripheral hydrogen-bonded ion pairs, TGFBR2 Asp^55^ with TGF-β3 Arg^394^ and TGFBR2 Glu^142^ with TGF-β3 Arg^325^, that further stabilise the interaction and add specificity are highlighted in yellow. TGF, transforming growth factor; TGM, transforming growth factor-β mimics.

A functional analysis of TGM1 in which amino (N)- and carboxy (C)-terminal domains were deleted showed that the minimal structure required for signalling through the TGF-β pathway comprises D1-3 [48]. Biophysical studies by surface plasmon resonance (SPR) and isothermal titration calorimetry established that D1-2 bind TGF-β receptor subunit I (TGFBR1) and D3 binds TGFBR2 [36] (Figure 1c). Mapping of the binding sites on TGFBR1 and TGFBR2 using nuclear magnetic resonance [36] further showed that D1-2 and D3 engage similar structural features and residues on these receptors as does the native ligand, TGF-β. Recently, a high-resolution (1.4 Å) structure of the D3-TGFBR2 complex (Figure 4a) revealed the remarkable mimicry of the binding interactions as compared with mammalian TGF-β, in spite of an entirely different structural framework (Figure 4b).

While domains D1-3 bind to the canonical receptors that are required for signalling, the function of D4 and D5 has only recently been clarified. Our findings indicated that TGM1 lacking D4 and D5 is considerably less potent than the full-length TGM1, suggesting that D4-5 are necessary for achieving the full potency of TGM1. This led us to speculate that D4 and D5 mediate cell specificity and potency by interacting with co-receptors. Indeed, TGM1 D4 and 5 have been found to interact with cell adhesion receptor CD44, which has a cell-type-restricted expression pattern and is essential for the potency of TGM1 [54] (Figure 1c). CD44 is an adhesion and migration molecule [55,56] widely expressed on immune cells and up-regulated on key subsets of memory/effector T cells [57], but is also found on endothelial and epithelial cells. In the context of immune cells, CD44 is required for efficient TGM1 activation, as shown both by reduced responses of CD44-deficient cells, and the loss of potency when D4-5 are removed from TGM1 and the remaining D1-3 is tested on fibroblasts or T cells [54]. Hence, it is suggested that TGM1 may have evolved to target CD44-expressing immune cells to dampen the host immune system during *H. polygyrus* infection; whether it also modulates epithelial cells such as CD44^+^ intestinal stem cells [58] remains to be determined.

Mammalian TGF-β homodimers have a compact structure that interacts with ubiquitously expressed TGFBR1 and TGFBR2 to assemble TGFBR1_2_:TGFBR2_2_ heterotetramers [59]. In contrast, TGM1 engages with TGFBR1, TGFBR2, through two independent modules (D1-2, D3) to form a TGFBR1:TGFBR2 heterodimer, and in association with a co-receptor through a third module (D4-5). For the biological activity induced by TGF-β1 and TGF-β3, the presence of TGFBR1 and TGFBR2 alone is sufficient. However, for TGF-β2, both TGFBR1 and TGFBR2 and the TGFBR3/betaglycan co-receptor are required [60,61]. TGFBR3 primes cells to respond to TGF-β2 by potentiating receptor complex assembly and signalling and is a key determinant for TGF-β2 cell specificity [44,62]. The way TGM1 binds to cell surfaces is, therefore somewhat similar to TGF-β2, as signalling requires the cooperation of three receptors. However, unlike TGF-β2 where the co-receptor TGFBR3 is displaced by the signalling receptors [44,63], TGM1 independently binds TGFBR1 through D1-2, TGFBR2 through D3 and remains bound to the co-receptor through D4-5 as it signals through TGFBR1 and TGFBR2.

## TGM co-receptor binding and cell specificity

Of the family of ten TGMs, TGM1–4 share a similar modular structure with five discrete domains, while TGM5, TGM6, TGM9 and TGM10 lack one or two domains, and TGM7 and TGM8 have acquired two additional domains (Figure 3a). The D1-3 of TGM1 that bind to TGFBR1 and TGFBR2 share high amino acid sequence identity (90–100%) with the corresponding domains of TGM2, 3, 4 and 5 but have lower sequence identity (50–66%) with those of TGM6–10. At the biophysical level, D3 of TGM1, 4 and 6 has each been shown to interact with TGFBR2 [36,49,50], and this suggests that all TGMs that include D3 may interact with TGFBR2, and those with D12 with TGFBR1.

**Figure 5:**
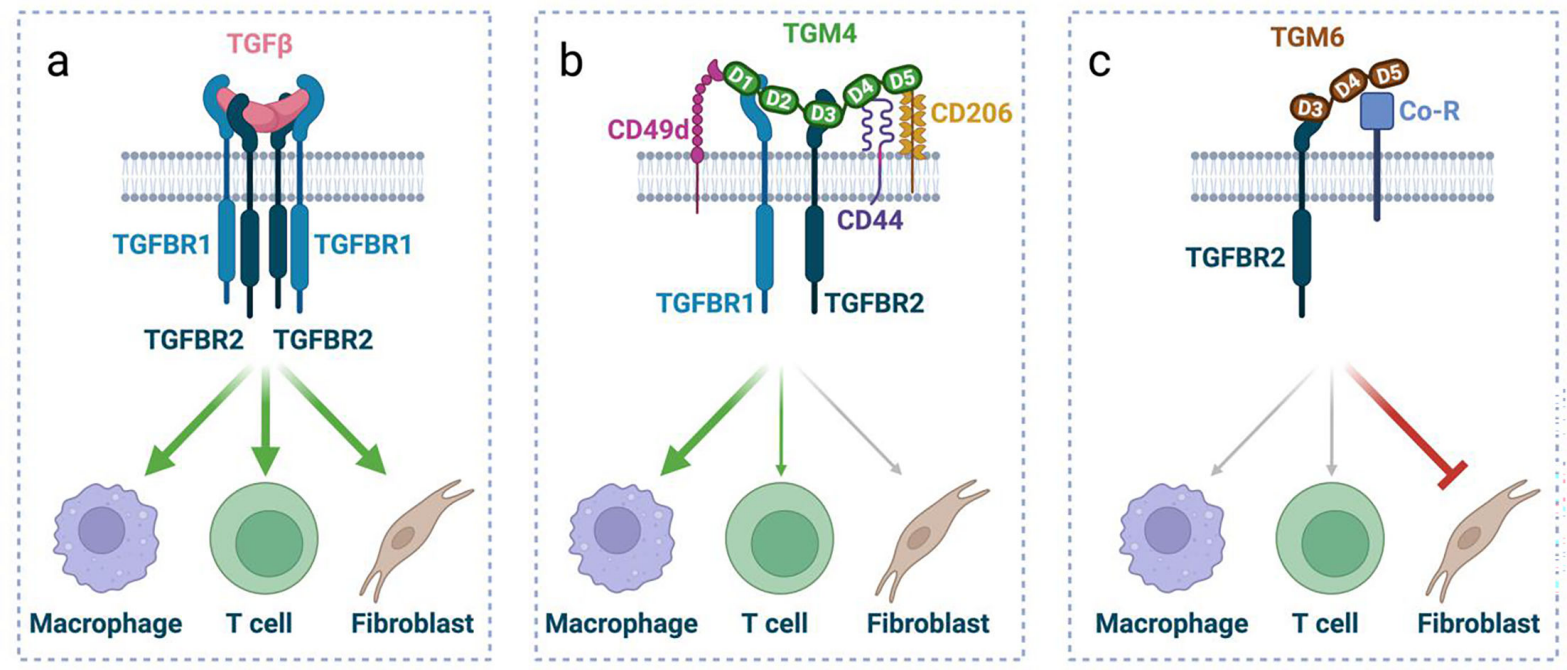
Model of cell specific actions of TGM proteins. Conceptual model comparing receptor interactions and cell specificity of TGF-β, TGM4 and TGM6. Created in BioRender. McSorley, HJ. (2025) https://BioRender.com/a55n320 (**a**) TGF-β acts as a homodimer to assemble a heterotetrameric complex of TGFBR1_2_:TGFBR2_2_ that signal to all cells expressing these receptors. (**b**) TGM4 ligates co-receptors including CD44, CD49d and CD206, while directly binding TGFBR1 and TGFBR2. D4-5 of TGM4 bind to CD44 and CD206; the domain binding to CD49d has not been conclusively identified. As a result of co-receptor interactions, TGM4 is selectively active on immune cells, particularly macrophages and does not activate fibroblasts with low or no expression of these co-receptors. (**c**) TGM6 lacks D1-2 and so cannot bind TGFBR1; it does however bind TGFBR2 together with a co-receptor (Co-R) that is different from those identified binding to TGM1 or TGM4. When added to cells expressing the appropriate co-receptor, such as fibroblasts, TGM6 acts as an antagonist of TGF-β signalling. TGF, transforming growth factor; TGM, transforming growth factor-β mimics.

The D4-5 of TGM1 are similar (87–99%) to D4-5 of TGM2, 3, 4 and 5 and are likely to share the same co-receptors. Indeed, we confirmed experimentally that TGM1 and TGM4 share CD44 as a co-receptor partner [49,54], but distinct from TGM1, TGM4 also interacts with CD49d (integrin α4) and CD206. Co-receptors confer cell specificity for TGF-β signalling as the individual affinity of TGMs for the TGFBRs can be moderate to low, such that the D3 of TGM1 and TGM4 binds TGFBR2 with affinities in the range of 1–2 and 50–100 μM, respectively [36,49]. The broader range of cell surface co-receptors for TGM4 compared with TGM1 results in increased cell specificity of TGM4 for monocytes rather than T lymphocytes or fibroblasts [49], prescribing a myeloid cell preference for TGM4 that may allow it to dampen immune activation without incurring the risk of fibrosis (Figure 5).

## Inhibitor discovery and diversification of TGMs in *H. polygyrus*

TGM6, 9 and 10 stand out for their absence of D1-2 which, in other family members, bind TGFBR1. We hypothesised that these proteins could act as antagonists if, for example, they sequestered TGFBR2 without recruiting TGFBR1to permit its phosphorylation. Indeed, TGM6 was recently found to retain and even have enhanced binding to TGFBR2 compared with TGM1 (*Kd* 1-2 μM and ~0.3 μM for TGM1 and TGM6, respectively) [36,50]. TGM6 also competed with TGF-β for binding TGFBR2, but it did not bind TGFBR1 or any other type I receptors of the TGF-β family. In reporter assays in mouse cells, such as MFB-F11 and NIH-3T3 fibroblasts, TGM6 was found to potently inhibit signalling by either TGM1 or TGF-β with sub-nanomolar potency [50]. However, TGM6 was unable to inhibit TGF-β signalling in some other cell types, such as splenic T cells. The latter, together with the inability of TGM6 to bind CD44, suggests that TGM6 may require a co-receptor, presumably engaged by D4-5, that is specific to fibroblasts and other populations such as epithelial cells that can also be inhibited by TGM6, in contrast with TGM4 which is specific for immune cells through a different co-receptor profile (Figure 5).

TGM6, 7, 8, 9 and 10 share lower sequence similarity (28–32%) with D4-5 of TGM1 and are likely to interact with different co-receptors than TGM1. Consistent with this notion, we found that TGM6 did not bind CD44 and antagonism by TGM6 depended on the physical attachment of D3 with D4-5 [50]. Our TGM (co)receptor identification studies, with both TGM agonists and antagonists, suggest that TGMs deliver TGF-β agonistic or antagonistic signals via in-cis interaction with ubiquitously expressed host TGF-β receptors and cell-type selective co-receptors. Therefore, identification of co-receptors for TGM6, 7, 8, 9 and 10 and the cell populations that express these receptors is eagerly awaited.

TGM1–10 are all produced by the same species, *H. polygyrus*. One possible reason for the secretion of multiple TGMs is that TGF-β is a highly contextual and multifunctional protein. Activating a TGM with similar binding and signalling characteristics to mammalian TGF-β might confer benefits, but it would also likely have adverse effects and thus offer no net benefit to *H. polygyrus*. To maximise their fecundity and longevity, the modular domain structure of the TGMs has enabled *H. polygyrus* to evolve the secretion of TGMs that specifically activate or inactivate TGF-β signalling in specific cell types (Figure 5). Our recent TGM binding studies have provided insights into how the cell-specific mechanism is achieved. The key point is that the affinities of TGMs D1-3 for signalling TGF-β receptors, particularly TGFBR2, are too weak to trigger TGF-β (agonistic) responses. For instance, TGM4 has more than a 100-fold lower affinity for TGFBR2 than TGF-β. Moreover, TGMs D1-3 must be physically connected to D4-5 (as they are in wildtype TGMs) to elicit TGF-β modulatory responses [36,49,50]. If cells are treated with a combination of TGM4–5 and truncated versions of TGM D1-3, TGM activity cannot be rescued. These findings suggest that binding TGM D4-5 to co-receptors is essential to increase cell surface avidity, above that accomplished solely by TGM D1-3 alone. The simultaneous combinatorial TGM interactions with (co-)receptors in-cis allow for the delivery of cell-specific specific responses.

## Evolutionary history

*H. polygyrus* enhances its survival in mouse hosts by exploiting the immune suppressive pathway mediated by TGF-β. It achieves this by having convergently evolved the functionally similar TGMs, which, despite primary amino acid sequences and structures being completely unrelated to that of mammalian TGF-β, are nonetheless able to interact with host TGFBRs. Phylogenetic analysis (Figure 3b) suggests that the three domain TGMs, TGM6, 9 and 10, are the ancestors of the family [51], indicating that D3 and its ligation to TGFBR2 was an ancestral characteristic. If correct, this suggests that TGMs likely evolved to antagonise the TGF-β pathway, perhaps as a mechanism to minimise fibrotic damage to the host as the parasite progresses through its life cycle in which the larvae bore though the intestinal epithelium to encyst in the muscle, and after maturation into adults, bore once again through the epithelium to the lumen to reproduce and for eggs to be excreted in the faeces.

Helminths are common parasites in humans and other animals, creating a notable interest in the presence of TGF-β mimetic proteins beyond the Heligmosomidae family, which includes *H. polygyrus* and species of the *Heligmosomum* genus. Some evidence suggests an archaic precursor in the ruminant nematode *Haemonchus contortus*, which belongs to the sister family Trichostrongylidae, both within the Strongyloidea superfamily [51]. The lack of TGMs beyond *H. polygyrus* is caused by a paucity of full-length cDNA sequences and incomplete whole genome sequences for species within and beyond its own taxonomic family. It would be valuable to address this gap and investigate the presence of TGMs in species other than *H. polygyrus*.

## True TGF-β homologues in helminths

The TGF-β family is well conserved: TGF-β encoding genes are identifiable across all metazoan organisms [64,65]. The model nematode *Caenorhabditis elegans* expresses five TGF-β family encoded genes, DAF-7, TIG-3, DBL-1, TIG-2 and UNC-129, the first two being most closely related to TGF-β [66,67]. *C. elegans* also possesses both type I (DAF-1, SMA-6) and type II (DAF-4) receptors, the latter even able to bind human BMP-2 and BMP-4 proteins [68]. In the best characterised system, the DAF4 receptor controls entry of *C. elegans* into arrested development, which is blocked in the presence of the TGF-β family ligand DAF-7 [69], while more recent studies have reported a remarkable involvement of DBL-1 and TIG-2 in the innate immune response of *C. elegans* to bacterial infection [67].

Subsequently, multiple TGF-β family members and their receptors have been identified across a wide range of parasitic nematodes [70–75], trematodes [76,77] and cestodes [78]. The presence of TGF-β family genes across parasitic and non-parasitic helminth species implies that these genes have a developmental role in all helminths. Interestingly, while DAF-7 prevents arrested development in *C. elegans*, the role of TGF-β signalling appears to be reversed in parasitic species [79]. Arrest is obligatory for larval parasitic stages until they take the opportunity to infect a new host, and in species such as the mosquito-borne *Brugia malayi*, expression of the TGF-β homologue TGH-2 is maximal in blood-stage microfilariae that await uptake by the arthropod vector before recommencing development [71,79]. More broadly, genetic knockdown of TGF-β family members in parasitic helminths has confirmed developmental requirements for many of these genes, with roles in trematodes in embryogenesis [76] and in the moulting and development of parasitic nematodes [75]. For example, a recombinant activin-like product of the liver fluke *Fasciola hepatica* enhanced parasite egg embryonation and parasite viability [80], while RNAi knockdown of the corresponding gene compromised the development and movement of newly excysted juvenile parasites [81].

Since this well-conserved family of proteins retains similarity to their mammalian counterparts, it would be logical to propose that gene duplication of parasite TGF-β family members, and evolution to bind to host TGF-β receptors could be an efficient mechanism of parasite immunomodulation [82]. Indeed, it has been reported that TGF-β homologues from *Brugia malayi* (Bm-TGH-2) [71] and *Fasciola hepatica* (FhTLM) [77] may signal via host TGF-β family receptors. However, when tested in T cell culture assays, no helminth homologue has been found to replicate the Foxp3^+^ regulatory T cell expansion activity of host TGF-β or TGM1 [78,81]. A TGF-β homologue from *Echinococcus multilocularis* (EmACT) has been reported that is only distantly related to human TGF-β, with 24% amino acid identity in the C-terminal receptor-binding region, albeit slightly higher similarity to activin. EmACT cannot directly induce Foxp3^+^ regulatory T cells in culture, but can amplify Foxp3 induction by low concentrations of recombinant TGF-β [78]. This effect is similar to that seen for mammalian activin A, which requires TGF-β and TGF-β-like receptor signalling to mediate its regulatory T cell-promoting effects [83].

Some unanswered questions remain about these interactions. For example, do endogenous TGF-β receptors in helminths recognise and respond to host-derived ligands, as suggested by a report that exposure of adult *S. mansoni* worms to human TGF-β induces a gene response that is blocked by knockdown of the parasite type II receptor [84]. Furthermore, the possibility that worm receptors can respond to the *H. polygyrus* TGMs remains to be explored.

Therefore, parasitic nematodes produce multiple TGF-β family proteins, and it is possible that some may be involved in modulation of the host immune response. Further investigations are required to identify which of these TGF-β family proteins are developmental, immunomodulatory or both and to ascertain their complementary TGF-β family receptors. More broadly, the presence of *bona fide* helminth TGF-β homologues poses the question of why *H. polygyrus* evolved the TGM family from a completely unrelated ancestor to carry out this immunomodulatory function.

## Other cytokine modulators from helminth parasites

The TGF-β pathway is clearly a rich seam for parasite immunomodulation, but it is far from the only cytokine signalling pathway modulated by parasite secreted factors [10]. Parasite products target many cytokines that either induce or suppress type 2 immune responses, including the interleukin (IL)-13, IL-33 and macrophage migration inhibitory factor (MIF) pathways.

IL-13 is a critical effector cytokine in the type 2 immune response: although pleiotropic, it is particularly effective on stromal cells, inducing goblet and tuft cell differentiation in the intestinal tract, together with smooth muscle hyperplasia. These cellular effects increase mucus secretion and peristalsis, mediating the ‘weep and sweep’ response for helminth ejection. In *T. muris* infection, IL-13 production is particularly key to resistance versus susceptibility [85], with IL-13-deficient mice on a resistant genetic background becoming highly susceptible to the parasite. Therefore, IL-13 seems a prime target for modulation by *T. muris*. Secretions of *T. muris* are dominated by a single protein, p43 [86], the gene for which is the tenth most highly expressed transcript in the adult parasite [87]. p43 binds directly to IL-13 and blocks the activity of this cytokine, inhibiting IL-13 responses during infection. Importantly, although p43 is not immunogenic during natural infection, vaccination with p43 in an alum adjuvant resulted in parasite ejection, thought to be due to the blockade of p43-mediated immunomodulation of IL-13 [86].

Responses to the alarmin cytokine IL-33 are also strongly implicated in immunity to helminths, with increased susceptibility of mice deficient in either IL-33 or the IL-33 receptor (ST2) seen in a wide range of helminth infections [4]. Therefore, as is the case for IL-13, the IL-33 pathway constitutes a crucial focus of helminth immunomodulation. *H. polygyrus* HES suppresses IL-33 responses in models of asthma [88,89] and in helminth infection [90] through down-regulation of ST2 transcription [89] and through inducing IL-1β release, which in turn suppresses production of IL-33 and IL-25 [90]. Finally, HES contains two families of proteins that interact directly with IL-33 or ST2, respectively. The *H. polygyrus* Alarmin Release Inhibitor (HpARI) family binds directly to IL-33 [91,92]. The HpARI family contains three members (HpARI1-3), of which HpARI1 and HpARI2 suppress responses to IL-33, while HpARI3 stabilises IL-33 and amplifies responses to the cytokine [93]. It is unclear why the parasite encodes proteins that have directly opposing functions; however, an explanation may stem from the finding that while HpARI1 and HpARI2 also bind to the extracellular matrix, HpARI3 does not [94], which could result in spatial or temporal separation of their effects *in vivo*. Finally, HES also contains the Binds Alarmin Receptor and Inhibits (HpBARI) family, containing HpBARI and HpBARI_Hom2 [95]. Both HpBARIs act similarly, binding with high affinity to ST2 and blocking interaction between the cytokine and its receptor. Thus, considerable evidence indicates that *H. polygyrus* applies significant resources to suppress IL-33 pathway, indicating the importance of this cytokine in resistance to the parasite.

Remarkably, HpARI and HpBARI have also both descended from an ancestral domain and are thus distantly related to the TGM family; it appears that within the *H. polygyrus* lineage, there has been repeated duplication and expansion of -like genes so that they are highly represented in the parasite genome [10]. Thus, while no direct homologues of the HpARIs or HpBARIs have been identified in other parasites (as is also the case for TGMs), there are multiple domains encoded in all helminth genomes and it appears likely that other parasites will have adapted these for the purposes of immune evasion in one manner or another [96].

The MIF pathway, as its name suggests, is important in the activation of macrophages. Its effects are highly context-dependent, activating macrophages in inflammatory settings but also having a role in the induction of type 2 immune responses via activation of a type 2 innate lymphoid cell (ILC2)-tuft cell pathway [97]. As host MIF expression is required for optimal anti-parasite immunity [98], it is surprising that many parasitic helminths secrete MIF homologues. Indeed, the MIF family is well conserved across many organisms, whether free-living or parasitic [99]. Several parasitic helminth MIFs show immunosuppressive properties via induction of anti-inflammatory macrophage activation (*Brugia malayi* Bm-MIF1/2 [100], induction of immunosuppressive cytokines (*Strongyloides ratti* Sra-MIF [101]) or regulatory T cell recruitment (*Anisakis simplex* As-MIF [102]). With an eye to the host agonism/antagonism via the TGM and HpARI families, it would be timely to revisit this work to test whether closely related MIF homologues are agonists versus antagonists and whether like the TGMs they modulate MIF signalling in a context-specific manner that would be optimal for the parasite.

## Other helminth products modulating host responses

Helminths also secrete many other molecules that heavily affect host immune responses and target towards myeloid immune cells, such as DCs and macrophages. A well-studied example is the enzyme ribonuclease, omega-1, secreted by eggs of *Schistosoma mansoni* [103]. Omega-1 primed DCs for Th2 polarisation [104,105] through binding of the mannose receptor via Lewis X glycan structures. Upon internalisation in DCs, protein synthesis was inhibited [106], while omega-1 also affected inflammasome-dependent IL-1-β in macrophages [107]. Experimental models showed that the administration of recombinant omega-1 *in vivo* targeted type 2 conventional DC (cDC2) and prevented allergic asthma by hampering DC migration [108]. This effect was dominant over the type 2 polarisation. Likewise, the fatty acid binding protein (FABP)1 from *F. hepatica* influenced DC function and T cell polarisation through the induction of thrombospondin-1 in DCs [109]. The molecule ES-62 is produced by the filarial nematode *Acanthocheilonema viteae* and has anti-inflammatory activities demonstrated in numerous models of inflammatory diseases [110]. ES-62 is a tetrameric glycoprotein, comprising of identical monomers of ~62 kDa and exerts its anti-inflammatory effect through a phosphorylcholine (PC)-containing moiety. ES-62 targets toll-like receptor (TLR)4 and affects downstream signalling leading to enhanced extracellular signal-regulated kinase (ERK)1/2 activation and DC hyporesponsiveness [111]. Apart from DCs, B cells are also targeted by secreted schistosome enzymes and molecules. For example, both recombinant glycoprotein antigen IPSE/α1, Sm14 and Thioredoxin-1 (SmTrx-1), induced IL-10-producing regulatory B cells, instrumental in driving regulatory T cells and immune evasion [112,113].

Interestingly, an increasing number of studies suggest that helminth-derived molecules impact metabolic activity and in that manner modify the function of specific immune cells, like macrophages. For example, it was suggested that recombinant omega-1 improved whole-body metabolic homeostasis [114,115]. Another class of molecules in this context form the cystatins, cysteine protease inhibitors which are produced by numerous helminth species, including *A. viteae, Ascaris lumbricoides, S. japonicum* and *S. mansoni*. Cystatin administration shows inhibitory effects in disease models [116–118]. This may be partially regulated by enhanced mevalonate and cholesterol biosynthesis pathways and immunomodulatory gene activity in DCs and macrophages, as demonstrated for cystatins secreted by *A. lumbricoides* [119]. An immunomodulating peptide secreted by *Fasciola hepatica,* named helminth defence molecule (FhHDM)1 was able to switch macrophage metabolism to oxidative phosphorylation fuelled by fatty acids and the induction of glutaminolysis. This was accompanied by decreased tumour necrosis factor and IL-6 production pointing to a role of switched immunometabolism in immune suppression [120]. Lastly, a recent study demonstrated that helminthic glutamate dehydrogenase secreted by *H. polygyrus* actively suppresses macrophage-mediated host defence through induction of prostaglandin E2 and suppression of type 2-promoting leukotrienes via 2-hydroxyglutarate (2-HG) [121]. Increased 2-HG is associated with reprogrammed cellular metabolism and is usually linked to hypoxia and mitochondrial dysfunction. 2-HG is grouped with other tricarboxylic acid cycle metabolites, such as acetyl-CoA, itaconate, succinate, fumarate, which are known for their capacity to alter both innate and adaptive immune cell systems through their ability to modify chromatin remodelling and DNA methylation, rewiring the epigenetic landscape of immune cells [122]. We are only at the beginning of understanding how such metabolic cues can induce long-term functional consequences for immune cells and how helminths have mastered the art of manipulating these processes through the secretion of enzymes and other interference mechanisms.

## Conclusions

The example of the helminth TGMs is instructive both in scope – that parasites can evolve unprecedented ligands that target pivotal pathways in host immunity – and in strategy, for combinatorial receptor binding is an especially effective means of selectively acting on specific host cell populations. As well as specificity, multivalency bestows avidity that will always exceed the affinity of any individual ligand-receptor pair. The multi-domain TGMs extend this further with trivalent interactions, an example with few parallels in extracellular receptor biology.

There are many fascinating nuances to be gleaned from this system: for example, the moderation of affinity for the canonical TGF-β receptors is key to the success of TGMs, as higher affinity would obviate the requirement for co-receptors and ablate cell specificity. Trivalency enables high avidity to be attained while maintaining individual receptor binding at low-to-medium affinity levels. Furthermore, maximal affinity does not always translate into optimal efficacy, as demonstrated in the setting of agonistic monoclonal antibodies for checkpoint immunotherapy, which act by driving a signal rather than blockade [123].

The interconversion of agonists and antagonists by gain or loss of binding modules represents a further quintessential feature of the TGM family. Mimicry to overturn function, as in the case of the antagonists, is not unique: the plant pathogen *Phytophthora* Suppressor of RNA silencing 2 protein PSR2 comprises divergent LWY modules, one of which mimics a host plant phosphatase subunit and combines with the remaining subunits to redirect its specificity to new protein targets [124].

In conclusion, TGM modularity offers exciting opportunities for exploitation in the setting of TGF-β signalling: if we can recombine, redesign and repurpose agonists and antagonists to different target cells and tissues, introducing novel modules with new specificities and fine-tuning affinities for maximum discriminatory properties, we may realise the therapeutic potential of this remarkable gene family across a wide range of disease settings.

## Open Access

For the purpose of open access, the authors have applied a Creative Commons Attribution (CC BY) licence to any Author Accepted Manuscript version arising from this submission.

## Abbreviations

CCP: complement control protein
TGF: transforming growth factor
TGMs: transforming growth factor-ß mimics
